# Supplementary material for: Clinical and molecular characteristics associated with high PD-L1 expression in EGFR-mutated lung adenocarcinoma
Source: PLoS One. 2024 Nov 7;19(11):e0307161. doi: 10.1371/journal.pone.0307161 (PMC11542846; doi:10.1371/journal.pone.0307161)
Supplement: S1 Table — (DOCX) [file pone.0307161.s002.docx]

| **PFS** | | | | | **OS** | | | |
| --- | --- | --- | --- | --- | --- | --- | --- | --- |
|  | **Univariate** | | **Multivariate** | | **Univariate** | | **Multivariate** | |
| **Characteristics** | **HR (95 % CI)** | **p-value** | **HR (95 % CI)** | **p-value** | **HR (95 % CI)** | **p-value** | **HR (95 % CI)** | **p-value** |
| **Age** (n=88) | 0.91 (0.24 – 3.47) | 0.90 |  |  | 1.53 (0.50 – 4.67) | 0.46 |  |  |
| **Gender** |  | 0.06 |  |  |  | **0.03** |  |  |
| Female (n=55) | 0.57 (0.33 – 1.01) |  |  |  | 0.53 (0.30 – 0.94) |  |  |  |
| Male (n=33) | 1.74 (0.99 – 3.01) |  | 1.96 (0.93 – 4.11) | 0.07 | 1.88 (1.07 – 3.31) |  | 2.05 (1.02 – 4.10) | **0.04** |
| **Smoking (pack-year)** | 1.23 (1.01 – 1.51) | **0.04** | 1.38 (1.06 – 1.79) | **0.01** | 1.35 (1.08 – 1.69) | **0.008** | 1.50 (1.12 – 1.95) | **0.005** |
| **PD-L1 ≥ 50%** (n=17) | 1.52 (0.7698 – 2.98) | 0.23 | 2.41 (1.14 – 5.10) | **0.02** | 1.71 (0.86 – 3.39) | 0.12 | 2.7 (1.23 – 5.97) | **0.01** |
| **ECOG :** 3 - 4 | 1.42 (1.04 – 1.95) | **0.03** | 1.52 (1.08 – 2.13) | **0.01** | 1.63 (1.15 – 2.3) | **0.006** | 2.09 (1.46 – 3.00) | **< 0.001** |
| **EGFR mutation** |  |  |  |  |  |  |  |  |
| Common (n=66) | 0.94 (0.49 – 1.80) | 0.84 |  |  | 0.63 (0.32 – 1.23) | 0.17 | 0.4 (1.8 – 0.91) | **0.03** |
| Rare (n=5) | 1.12 (0.35 – 3.68) | 0.83 |  |  | 0.96 (0.30 – 3.12) | 0.95 | 0.26 (0.06 – 1.09) | 0.06 |
| Complex (n=11) | 1.62 (0.75 – 3.47) | 0.22 |  |  | 2.45 (1.23 – 5.31) | **0.02** |  |  |
| Ins exon 20 (n=6) | 0.27 (0.04 – 1.97) | 0.20 |  |  | 0.60 (0.08 – 4.38) | 0.61 |  |  |
| **Co-mutation (n=51)** | 1.00 ( 0.56 – 1.79) | 0.99 | 0.57 (0.30 – 1.08) | 0.08 | 0.90 (0.50 – 1.60) | 0.71 | 0.55 (0.30 – 1.03) | 0.06 |
| TP53 (n=42) | 1.37 (0.77 – 2.41) | 0.28 |  |  | 1.18 (0.67 – 2.10) | 0.57 |  |  |
| CTNNB1 (n=8) | 0.48 (0.14 – 1.45) | 0.18 |  |  | 0.45 (0.51 – 1.60) | 0.18 |  |  |
| **Metastasis** |  |  |  |  |  |  |  |  |
| Liver (n=22) | 4.07 (2.21 – 7.5) | **< 0.001** | 10.97 (4.86 – 24.80) | **< 0.001** | 3.02 (1.65 – 5.5) | **< 0.001** | 7.38 (1.25 – 15.96) | **< 0.001** |
| CNS (n=28) | 2.50 (1.40 – 4.47) | **0.002** | 3.21 (1.71 – 6.03) | **< 0.001** | 1.9 (1.08 – 3.4) | **0.03** | 2.33 (1.25 – 4.35) | 0.008 |
| Bone (n=48) | 1.82 (1.01 – 3.27) | **0.05** |  |  | 1.52 (0.85 – 2.70) | 0.16 |  |  |

**S1 Table. Univariate and multivariate analysis of progression-free survival and overall survival in 88 patients with smoking pack-years information available.** CNS:Central nervous system ; ECOG : eastern cooperative oncology group performance scale.
