# Supplementary material for: Clinical and molecular characteristics associated with high PD-L1 expression in EGFR-mutated lung adenocarcinoma
Source: PLoS One. 2024 Nov 7;19(11):e0307161. doi: 10.1371/journal.pone.0307161 (PMC11542846; doi:10.1371/journal.pone.0307161)
Supplement: Attachment — Submitted filename: Reponse to reviewer.docx [file pone.0307161.s004.docx]

Jun the 15th, 2024

Editor

PLOS ONE

Dear Dr Yamagushi,

Thank you for considering our manuscript titled "Clinical and Molecular Features Associated with High PD-L1 Expression in EGFR-Mutated Metastatic Lung Adenocarcinoma" (PONE-D-24-01682). We have diligently revised the text and tables in response to the reviewers' comments (detailed responses provided below). We believe these revisions have significantly enhanced the quality of our manuscript.

We have reviewed our reference list to ensure it is complete and correct

Yours sincerely,

Professor Marie Wislez, on behalf the coauthors

Corresponding author:

Professor Marie Wislez Pneumology, Cochin Hospital Paris, Université Paris Cité, Faculty of Medicine, Paris, France;

telephone: 33 158413091

e-mail: [marie.wislez@aphp.fr](mailto:marie.wislez@aphp.fr)

Subject: Reponse to Reviewers - "Clinical and molecular features associated with high PD-L1 expression in EGFR-mutated metastatic lung adenocarcinoma"

Reviewer #2: The authors have responded to my review comments by adding some analytical results, demonstrating that they do not affect the original conclusions of the authors. However, toxicities by EGFR-TKI according to PD-L1 expressions had not been presented in the revised manuscript because they do not have representative data. This should be noted in the discussion as a limitation.

**Thank you for your feedback. Indeed, this is a limitation that we did not address in the text. We have therefore added the following sentence: "Given the retrospective nature of this study, the analysis of the side effects of the different treatments received, according to PD-L1 expression, could not be cataloged and analyzed in this article, which represents a potential bias to consider when interpreting these results" on page 15, line 315-318.**

________________________________________________________________________________

Reviewer #3: my comments had been addressed well by the authors.

**We thank you for your review and your various comments.**

________________________________________________________________________________

Reviewer #4: The points raised by all the reviewers were adequately addressed by the authors. I have no further comments.

**We thank you for your review and your various comments.**
